# The E-Protein Daughterless Regulates Olfactory Learning of Adult *Drosophila melanogaster*

**DOI:** 10.1523/ENEURO.0051-25.2025

**Published:** 2026-01-20

**Authors:** Laura Tamberg, Carl Sander Kiir, Jürgen Tuvikene, Käthy Rannaste, Mari Palgi, Indrek Koppel, Tõnis Timmusk

**Affiliations:** ^1^Department of Chemistry and Biotechnology, Tallinn University of Technology, Tallinn 12618, Harjumaa, Estonia; ^2^Protobios LLC, Tallinn 12618, Harjumaa, Estonia

**Keywords:** ChIP sequencing, Daughterless, *Drosophila melanogaster*, E-protein, memory, transcriptomics

## Abstract

Daughterless (Da), the *Drosophila melanogaster* homolog of mammalian E-protein transcription factor 4 (TCF4), is well studied in fruit fly embryonic development but its functions in adult nervous system are poorly understood. Mutations in human *TCF4* gene lead to intellectual disabilities such as Pitt–Hopkins syndrome and *TCF4* has also been linked to schizophrenia. Here, to explore the roles of Da in the *Drosophila* mature brain, we map Da DNA binding sites and study the transcriptomics of the brains where Da function is inhibited by pan-neuronal Extramacrohaete (Emc) overexpression, in both male and female *Drosophila*. Our transcriptome analyses reveal that in the adult brain Da regulates the expression of genes involved in behavior, memory, synaptic signaling, protein translation, and metabolic processes. Moreover, combining the RNA sequencing data with Da ChIP sequencing results indicates that genes associated with neuronal projection guidance, metabolism, and translation are direct targets of Da. In addition, we validate the involvement of Da in memory formation. Overall, our results provide valuable information about the functions of Da in the adult brain and aid in better understanding the mechanisms of TCF4-related disorders.

## Significance Statement

Daughterless (Da), the *Drosophila melanogaster* homolog for mammalian E-proteins transcription factor 4 (TCF4), TCF3, and TCF12, is a basic helix–loop–helix transcription factor known for its roles during nervous system development. We have previously shown that Da is expressed in the adult *Drosophila* brain, but little is known of its functions there. TCF4, a human ortholog of Da, is associated with the intellectual disability syndrome Pitt–Hopkins syndrome and schizophrenia. The molecular mechanisms of these serious conditions are largely unknown. Investigating the functions of Da in the nervous system potentially provides information about the functioning of TCF4 as well and could help in better understanding the mechanisms of the diseases associated with TCF4.

## Introduction

Daughterless (Da) is a Class 1 basic helix–loop–helix (bHLH) transcription factor in *Drosophila melanogaster*, and it is homologous to three mammalian E-proteins—transcription factor 4 (TCF4), TCF3, and TCF12 (C. Murre et al., 1989; Massari and Murre, 2000; Tamberg et al., 2015). Understanding the roles of Da in the nervous system is important because its human ortholog, E-protein TCF4, is implicated in various nervous system disorders—mutations in *TCF4* lead to an autism spectrum disorder Pitt–Hopkins syndrome (PTHS), and its common gene variants are linked to schizophrenia (Amiel et al., 2007; Brockschmidt et al., 2007; Zweier et al., 2007; Doostparast Torshizi et al., 2019). Like its human homologs, Da forms homodimers or heterodimers with Class 2 bHLH proteins and binds to the Ephrussi box (E-box) motif on DNA (CANNTG) to activate transcription (Cornelis Murre et al., 1989; Cabrera and Alonso, 1991). In addition, Da heterodimerizes with Extramacrohaete (Emc), a *Drosophila* homolog of the mammalian inhibitor of differentiation (Id) protein, which negatively regulates the activity of Da through heterodimerization (Ellis et al., 1990; Van Doren et al., 1991; Cabrera et al., 1994; Spratford and Kumar, 2015; Waddell et al., 2019).

Da has been shown to be expressed in the *Drosophila* nervous system throughout the development. More precisely, it is expressed widely during embryogenesis with the highest levels in the developing nervous system (Cronmiller and Cummings, 1993; Vaessin et al., 1994). During the third instar larval stage, the expression levels of Da are high in the imaginal discs, salivary glands, and central nervous system (CNS; Cronmiller and Cummings, 1993; Tamberg et al., 2020). In adult flies, Da has been shown to be expressed in the reproductive system and in the CNS (Cronmiller and Cummings, 1993; Tamberg et al., 2020).

Da has many known functions during development, including sex determination (Cline, 1988; Deshpande et al., 1995; Hoshijima et al., 1995), cell cycle regulation (Andrade-Zapata and Baonza, 2014), endoderm development (Tepass and Hartenstein, 1995), mesoderm development and myogenesis (Castanon et al., 2001; Wong et al., 2008), and oogenesis (Cummings and Cronmiller, 1994; Smith et al., 2002) but is primarily known for its crucial role in embryonic nervous system development. In *da null* mutant embryos, the peripheral nervous system (PNS) is completely absent, and the CNS exhibits severe defects (Caudy et al., 1988; Tamberg et al., 2015). Ubiquitous overexpression of Da causes the development of ectopic neuronal cells (Giebel et al., 1997). In addition, in the larval brain, Da is required for neuroblast differentiation (Neumüller et al., 2011; Yasugi et al., 2014, 2008). Da is also important for the development of adult PNS. Da functions in the third instar larval eye imaginal discs during eye development (Brown et al., 1996; Chen and Chien, 1999; Cadigan et al., 2002; Sukhanova et al., 2007; Lim et al., 2008; Bhattacharya and Baker, 2011; Tanaka-Matakatsu et al., 2014; Wang and Baker, 2015; Li and Baker, 2019; Nair and Baker, 2024; Reddy Onteddu et al., 2024) and its crucial role during the development of wing, thorax, and leg sensory bristles (Jafar-Nejad et al., 2006; Sukhanova et al., 2007; Bhattacharya and Baker, 2011; Tamberg et al., 2015) has been extensively investigated.

In addition to being crucial for the development of the *Drosophila melanogaster* nervous system, Da is also important for the functioning of larval nervous system. Silencing of Da in the larval mushroom body impairs appetitive associative learning (Tamberg et al., 2020), and Da is implicated in synaptogenesis of the larval neuromuscular junctions (D’Rozario et al., 2016). Decreased levels of Da in the larval nervous system lead to misexpression of synaptic proteins (D’Rozario et al., 2016; Tamberg et al., 2020). Evidence also suggests involvement of Da in the adult fruit fly function—silencing of *da* in the adult *Drosophila* brain affects negative geotaxis of the flies and ubiquitous overexpression of Da after eclosion from the pupae results in death within days (Tamberg et al., 2020, 2015).

Although Da has been extensively investigated in the development of *Drosophila* nervous system, its roles in the adult brain are poorly understood. Here, we employed ChIP sequencing (ChIP-seq) and transcriptomics experiments to investigate downstream genes of Da. Our findings suggest that Da regulates genes involved in synaptic signaling, memory, metabolism, and protein translation in adult *Drosophila* brains. In addition, we used appetitive associative learning assay to further investigate the role of Da in adult memory formation.

## Materials and Methods

### Drosophila stocks

All *Drosophila* stocks and crosses were fed with malt and semolina-based food with 12 h light and dark daily rhythms at 25°C with 60% humidity. *Drosophila* strains used in this study were UAS-emc (FlyORF, F001792), UAS-da^G^ (BDSC, 37291), elavC155-Gal4 (BDSC, 458), white* (France Fly Facility), UAS-da^RNAi^ KK105258 (Vienna Drosophila Resource Center), UAS-Dcr2 (BDSC, 24644), nSyb-Gal4 (BDSC, 51941), ts-Gal80 (BDSC, 7016), UAS-nlsGFP (BDSC, 4776), and 3xFLAG-Da (Tamberg et al., 2020). The following transgenic lines were generated in this study: min-Luc and 12xE-box-Luc.

### Sample preparation for sequencing

RNA from elavC155 > emc or elavC155/+ 0–24-h-old adult *Drosophila* brains (175 males and 175 females per replicate) was isolated using RNeasy Mini Kit (Qiagen) according to the manufacturer's protocol. RNA integrity and concentration were analyzed using Agilent 2100 Bioanalyzer. Unstranded poly(A^+^) library preparation and paired-end 2 × 150 bp sequencing were performed at Novogene Europe.

Chromatin preparations for ChIP-seq were carried out as described previously (Chanas et al., 2004; Tamberg et al., 2020) from 1–3-d-old 3xFLAG-Da adult heads (about half of the heads male and half female). As a control, white* *Drosophila* line was used with no *FLAG* sequence in the genome. The heads were collected on dry ice and homogenized in buffer A1 [60 mM KCl, 15 mM NaCl, 4 mM MgCl2, 15 mM HEPES, 0.5% Triton X-100, 0.5 mM DTT, and 1× EDTA-free protease inhibitor cocktail (Roche)], pH 7.6, with 1.8% formaldehyde at room temperature using a Kontes pellet pestle followed by three strokes using a Dounce homogenizer (Wheaton) with a loose pestle. Homogenate was incubated for 15 min, and glycine was added to 225 mM final concentration followed by 5 min incubation at room temperature. The homogenate was then centrifuged for 5 min at 4,000 × *g* at 4°C, and the supernatant was discarded. The pellet was washed three times with 3 ml of buffer A1, followed by a wash with 3 ml of lysis buffer [14 mM NaCl, 15 mM HEPES, 1 mM EDTA, 0.5 mM EGTA, 1% Triton X-100, 0.5 mM DTT, 0.1% sodium deoxycholate, 0.05% SDS, 10 mM sodium butyrate, and 1× EDTA-free protease inhibitor cocktail (Roche)], pH 7.6. Cross-linked material was resuspended in 0.5 ml of lysis buffer with 0.1% SDS and 0.5% *N*-lauroylsarcosine and incubated for 10 min at 4°C on a rotator. DNA was sonicated using Diagenode Bioruptor Pico sonicator for 60 cycles at 60 s ON/60 s OFF intervals. Cross-linked material was then rotated for 10 min at 4°C and centrifuged for 5 min at 20,000 × *g*. Supernatant was then transferred to a new tube, and 0.5 ml of lysis buffer was added to the pellet followed by rotation and centrifugation as described above. Supernatants were combined and centrifuged at maximum speed two times for 10 min. Chromatin extract was transferred to Microcon DNA Fast Flow Centrifugal Filter Units (Merck Millipore), blocked with 1 mg/ml bovine serum albumin in PBS, and purified using lysis buffer. The volume of chromatin extract was brought to 1 ml using lysis buffer. Protein concentrations were determined using BCA Protein Assay Kit (Pierce). After taking equal amounts of inputs, chromatin extracts were diluted 10× using dilution buffer [1% Triton X-100, 150 mM NaCl, 2 mM EDTA (pH 8.0), 20 mM Tris–HCl (pH 8.0), and 1× EDTA-free protease inhibitor cocktail (Roche)] and added to 50 μl of Dynabeads Protein G (Invitrogen) beads that had been prebound with 5 μg of monoclonal anti-FLAG M2 antibody (Sigma-Aldrich F1804) in 400 μl of 0.05% PBS + Tween 20 overnight. Lysate was incubated with beads overnight at 4°C. Beads with chromatin were then washed in wash buffer [1% Triton X-100, 0.1% SDS, 150 mM NaCl, 2 mM EDTA (pH 8.0), 20 mM Tris–HCl (pH 8.0), and 1× EDTA-free protease inhibitor cocktail (Roche)] for 10 min for three times at 4°C on a rotator, followed by final wash with final wash buffer [1% Triton X-100, 0.1% SDS, 500 mM NaCl, 2 mM EDTA (pH 8.0), 20 mM Tris–HCl (pH 8.0), and 1× EDTA-free protease inhibitor cocktail (Roche)]. Chromatin was eluted two times using 50 μl elution buffer (1% SDS, 100 mM NaHCO3 and 1 mM EDTA) for 10 min each time at 37°C and one time for 10 min at 65°C. The volume of inputs was brought to 150 μl with elution buffer. For decrosslinking, 8 μl of 5 M NaCl was added and the samples were incubated at 65°C overnight. Then, 2 μl of RNase A (10 mg/ml) was added, and the samples were incubated at 37°C for 30 min, followed by the addition of 2 μl of EDTA (0.5 M) and 4 μl Proteinase K (10 mg/ml) and incubation at 45°C for 30 min. DNA was extracted using a QIAquick PCR Purification Kit (Qiagen). Library preparations and 75 bp single-end sequencing were performed at LGC Genomics.

### Bioinformatical analysis

RNA sequencing (RNA-seq) and ChIP-seq adapter and quality trimming were performed using BBDuk (part of BBMap version 38.90, sourceforge.net/projects/bbmap/) with the following parameters: ktrim = r k = 23 mink = 11 hdist = 1 tbo qtrim = lr trimq = 10 maq = 10 minlen = 25 for RNA-seq and minlen = 50 for ChIP-seq.

*Drosophila melanogaster* ChIP-seq reads were mapped to BDGP6.32 (primary assembly and annotation obtained from Ensembl, release 104, BDGP6.32) using Bowtie2 (version 2.5.1; Langmead and Salzberg, 2012). Resulting SAM files were processed with Samtools (version 1.12; Danecek et al., 2021) as follows: quality filtered (-q 20), sorted, removed duplicates (-s), and converted to BAM and indexed. Model-based analysis of the ChIP-Seq (MACS2, version 2.2.7.1; Zhang et al., 2008) tool was used to detect peaks in IP samples by comparing with corresponding inputs using the following parameters: -B -q 0.05 -s 75. The results were further analyzed with DiffBind (version 3.8.4; Stark and Brown, 2011; Ross-Innes et al., 2012), where peaks were centered to ±200 bp around detected summits to determine peak enrichment over control (signal in white*) and then visualized using ChIPseeker (version 1.34.1; Wang et al., 2022).

To summarize the ChIP peak enrichment relative to transcription start sites (TSS) and transcription end sites (TES), we merged biological replicate bam files and rerun MACS2. MACS2 output bedGraph files (merged IP sample signal intensity was normalized to corresponding merged input) were converted to bigWig format using ucsc-bedgraphtobigwig (version 377; Kent et al., 2010) and then processed using deepTools (version 3.5.0; Ramírez et al., 2016). computeMatrix command from deepTools was used to calculate scores per genome region by using the merged bigWig files and BDGP6.32 annotation with the following parameters: -b 2,000 -a 2,000. The resulting score matrix was visualized using plotProfile command.

To determine the DNA binding sequences, we used motif-based sequence analysis tool MEME Suite (version 5.5.1; Machanick and Bailey, 2011). Fasta sequence file was generated using a custom R script, where coordinates were taken from statistically significant peaks (compared with white*) from the DiffBind pipeline, which were classified as log_2_ fold change ≥ 1 and FDR ≤ 0.05. The resulting sequences were analyzed using MEME-ChIP with the following parameters: meme-chip -meme-norand -meme-nmotifs 10 -streme-nmotifs 10 -meme-searchsize 0 -ccut 100. To manually determine the prevalence of different E-box–containing motifs, all possible E-box patterns (CANNTG variants) were counted inside ChIP the peak regions (±50 bp of Da ChIP peak summits), and the control region set combined ±50 bp summit regions after shifting peaks either 250 bp upstream or downstream. Logistic regression analysis was performed for each unique E-box sequence using the glm function from R, and the results were visualized together with the proportion of detected E-box motifs inside and outside the ChIP peak regions. *P* value adjustment was applied using the Benjamini–Hochberg (BH) procedure.

*Drosophila melanogaster* RNA-seq reads were mapped to BDGP6.32 genome using STAR aligner (version 2.7.4a; Dobin et al., 2013) with default parameters. To increase sensitivity for unannotated splice junctions, splice junctions obtained from the first pass were combined per dataset and filtered as follows: junctions on noncanonical intron motifs were removed; only junctions detected in at least two samples (10% of samples rounded up to the nearest integer) in the whole dataset were kept. Filtered junctions were added to the second pass mapping using STAR aligner. RNA-seq reads were assigned to features using FeatureCounts (version 2.0.1; Liao et al., 2014). The following parameters were used for paired-end RNA-seq data: -p –B –C –J. The produced raw read counts were analyzed with DESeq2 (version 1.38.3; Love et al., 2014) to determine differentially expressed genes.

Gene Ontology (GO) analysis was performed using clusterProfiler (version 4.7.1.003; Wu et al., 2021) and ReactomePA (version 1.42; Yu and He, 2016) using differentially expressed genes. For RNA-seq, statistically significant genes were classified as *p*-adjusted values ≤ 0.05, with counts ≥20 in control (elavC155/+) or Emc^OE^(elavC155 > emc) samples; upregulated and downregulated genes were distinguished with log_2_ fold change ≥ 0.2 or ≤ −0.2, respectively. GO for Emc^OE^ RNA-seq and common genes in RNA-seq and ChIP-seq were done against a custom background based on our sequencing data, where background was defined as genes with counts ≥20 at least in control or Emc^OE^ samples. GO enrichment analysis was done with clusterProfiler and ReactomePA using the following parameters: *p* and *q* value cutoff <0.05, minimal gene set size 50, maximal gene set size 500, and *p* value adjustment method done with BH procedure.

The results were visualized using ggplot2 (version 3.4.1; Wickham, 2016), enhancedVolcano (version 1.16; Blighe, 2024), and VennDiagram (version 1.7.3; Chen, 2022) in R (version 4.1.2). RNA-seq and ChIP-seq tracks were visualized using Integrative Genomics Viewer (version 2.15.2; Robinson et al., 2011).

### Generation of 12xE-box reporter fly lines

12xE-box-Luc construct was created using oligonucleotides Sense, CTAGAGATCTGAACAGCTGCAAGAACAGCTGCAAGAACAGCTGCAAG, and Antisense, GATCCTTGCAGCTGTTCTTGCAGCTGTTCTTGCAGCTGTTCAGATCTCTAGAGCT containing three CAGCTG E-boxes (Microsynth). Oligonucleotides were kinated with T4 polynucleotide kinase A (Thermo Fisher Scientific) in T4 ligation buffer, annealed and subsequently inserted into a donor vector pGL3-Basic (Promega #E1751) four times using SacI and BglII restriction enzymes before cloning the 12xE-box sequence into the reporter construct pPTluc which contains a *Drosophila* minimal promoter (Addgene #87789). Reporter constructs with or without 12xE-box sequences were sent to Bestgene for creation of reporter fly lines.

### In vivo luciferase reporter assay

The 0–24-h-old fly brains (seven males and seven females) were dissected and lysed in Passive Lysis Buffer (Promega). Protein concentration was measured using a bicinchoninic acid assay (Pierce). Equal volume of Dual-Glo luciferase reagent (Pierce) was added to the lysates, and luminescence was measured. The data were then log transformed and auto scaled, means and standard deviations were calculated, and paired two-tailed Student's *t* tests were performed. The data were back-transformed to linear scale for graphical representation, and fold over control was shown.

### Appetitive associative learning of adult flies

Learning paradigm was modified from Malik and Hodge (2014). The 0–24-h-old control and Emc^OE^ or da^RNAi^ flies (15–30 flies, about half males and half females) were starved for 16 h at 25 or 18°C where specified in a vial containing moist sponge. Flies were transferred without anesthesia to a training vial containing a blotting paper previously soaked with deionized water and dried. The vial was attached to a *T*-maze (Maze Engineers) and left to rest while air flow was applied for 30 s. Then, first odor presented. After 2 min, the odor was removed, and the flies were left to rest for 30 s while air flow was applied. Next, the flies were transferred to the other training vial containing a blotting paper previously soaked with 2 M sucrose and dried, and the other odor was presented for 2 min. Then the flies were tapped to the resting chamber, and the maze was prepared for test—one odor was attached to one arm and the other to the other arm of the maze. After 90 s resting, the flies were pushed to the test arms using the elevator and left to move freely for 2 min. Then, the flies were tapped to vials and counted. Next, reciprocal training was conducted and performance indexes calculated. The odor represented first during training and the sides of the odors during the test were alternated every time. The odor-sensing controls were done according to Malik and Hodge (2014). For sucrose sensing controls, the flies were starved similarly to the memory test.

### qPCR

For RT-qPCR, RNA from 10 1-d-old *Drosophila* (half males and half females) brains was extracted using RNeasy Mini Kit (Qiagen). cDNA was synthesized with Superscript IV Reverse Transcriptase (Invitrogen) and oligo-dT20 primers. qPCR was performed using a LightCycler 480 II (Roche) with Hot FIREPol EvaGreen qPCR Mix Plus (Solis Biodyne) using emc primers (GAAAGTCTCTATCCCGCCG and CAAGAGTGTTGGGCGTTTGG) and for normalization alpha-tubulin primers (TGGGCCCGTCTGGACCACAA and TCGCCGTCACCGGAGTCCAT).

### Ex vivo protein labeling with puromycin and Western blotting

Fly brains (seven males and seven females) were dissected in PBS and transferred to *Drosophila* hemolymph like medium (108 mM NaCl, 5 mM KCl, 2 mM CaCl_2_, 8.2 mM MgCl_2_, 4 mM NaHCO_3_, 1 mM NaH_2_PO4, 5 mM trehalose, 10 mM sucrose, 5 mM HEPES), pH 7.5, containing 5 µM puromycin (Cabrera-Cabrera et al., 2023). The labeling was done in 12-well tissue culture plates containing 800 µl of the medium for 1 h at 25°C. The brains were then transferred to 2× Laemmli buffer containing 10% β-mercaptoethanol and heated for 10 min at 95°C and sonicated with Diagenode Bioruptor Pico sonicator for 30 s ON/30 s OFF for three cycles. Equal amounts of lysate were loaded to 10% SDS-PAGE gel and transferred to PVDF membranes using Trans-Blot Turbo Transfer system (Bio-Rad Laboratories). Anti-puromycin (Millipore MABE343, 1:2,000) and anti-mouse IgG-HRP 32430 (Thermo Fisher Scientific) were used for immunoprobing.

### Immunohistochemical staining of adult *Drosophila* brains

Adult flies were first fixed in 4% paraformaldehyde in PBS and then dissected. Primary antibody labeling was performed for 72 h with the mouse anti-FLAG M2 (dilution 1:1,000; Sigma-Aldrich F1804) antibody while gently shaking at 4°C in PBS using 0.5% TritonX-100. Secondary antibodies goat anti-mouse Alexa Fluor 594 (dilution 1:1,000; Jackson ImmunoResearch Laboratories 115-585-003) were preadsorbed to wild-type tissues before use. Incubation with secondary antibodies was performed for 3 h at room temperature in PBS with 0.1% Triton X-100. The labeled larval brains were dissected and mounted in Vectashield mounting medium (Vector Laboratories). For image collection, Zeiss LSM 900 confocal microscope with a Plan- Apochromat 20× (NA 0.8) objective was used. Suitable layers were selected using Zeiss ZEN 3.12 software.

## Results

### Daughterless binds to regions near TSS and prefers CAGCTG E-box in adult *Drosophila* heads

**Figure 1.**
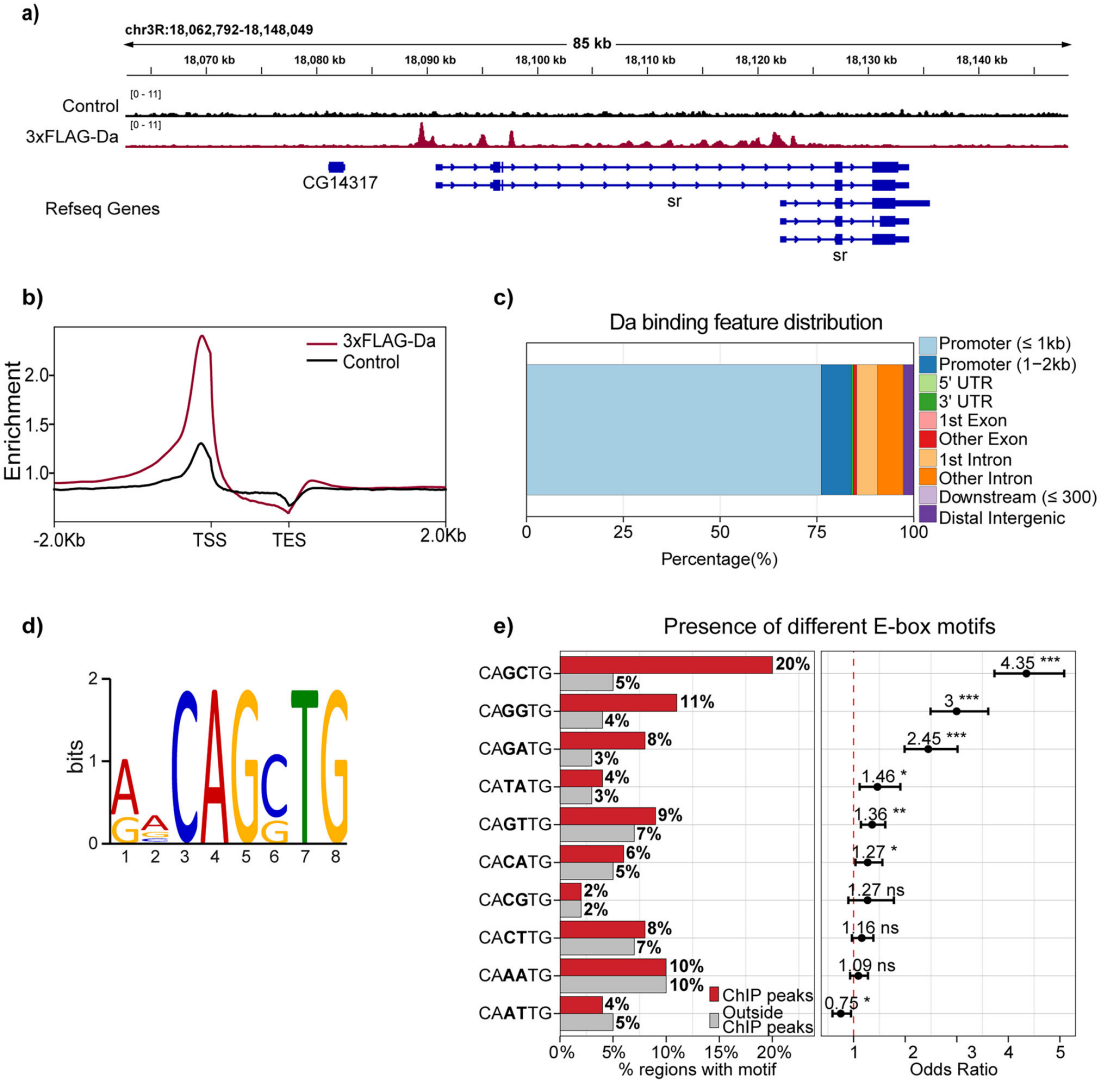
Genome-wide analysis of Da binding sites by ChIP-seq. ***a***, Visualization of anti-FLAG ChIP-seq data from adult *Drosophila* heads of 3xFLAG-Da and control (white*) flies. Data were visualized as fold over input, and for graphical visualization two biological replicates were merged. As an example, *stripe (sr)* gene locus is shown, where 3xFLAG-Da protein binds to the promoter regions and several regions in the introns. ***b***, Distribution of anti-FLAG ChIP peak enrichment relative to TSS and TES. Replicates were merged for visualization. ***c***, Genomic distribution of 3xFLAG-Da protein binding sites. ***d***, MEME-ChIP was used to find transcription factor-binding motifs; E-box sequences CAGCTG and CAGGTG were the most significant. ***e***, The occurrence of all possible E-box motif variants (CANNTG) within ±50 bp of Da ChIP peak summits and in 250 bp shifted control regions. The control region set combines ±50 bp regions after shifting either 250 bp upstream or downstream. Left, Proportions of detected variant E-box motifs (variant NN nucleotides are shown in bold) within the Da ChIP peaks (red) and control regions (gray). Right, Odds ratios from logistic regression analysis comparing E-box presence at Da ChIP peaks versus control regions. Black circles represent the odds ratio (exponent of estimate), and horizontal bars indicate 95% confidence intervals. The red dashed line marks an odds ratio of 1 (no enrichment or depletion). ***p < 0.001; **p < 0.01; *p < 0.05; ns = not significant; BH procedure-adjusted *p* values. Significantly enriched peaks in 3xFLAG-Da samples compared with white* samples are listed in Extended Data Figure 1-1.

In order to elucidate the roles of Da in the adult *Drosophila* nervous system by investigating its target genes, we first decided to identify Da binding to the genome in fruit fly heads using ChIP-seq. We took advantage of a *Drosophila* line previously generated by us where endogenous Da is tagged with 3xFLAG (Tamberg et al., 2020). This tagged protein, 3xFLAG-Da, retains its functionality, as demonstrated in our earlier study using a luciferase reporter assay (Tamberg et al., 2020). For the control, we used white* fly line with no FLAG tag in the genome, because the 3xFLAG-Da line was originally generated into the white* background (Tamberg et al., 2020). The ChIP-seq revealed 2,553 regions that were significantly enriched in 3xFLAG-Da samples compared with the control samples (Fig. 1*a*; Extended Data Fig. 1-1), corresponding to 2,126 genes. A detailed analysis of binding-site distribution showed that Da binds preferentially to promoter regions, with ∼75% of the peaks located within promoter regions, <1 kb from the TSS (Fig. 1*b*,*c*). In addition, a substantial number of peaks were observed within 1–2 kb distance of promoters, in first introns and other intronic regions. Motif analysis of 3xFLAG-Da binding sites revealed that Da binds preferably to CAGCTG, CAGGTG, and CAGATG E-box sequences in the adult fly heads, most preferred being the CAGCTG E-box (Fig. 1*d*,*e*). Collectively, the analysis of 3xFLAG-Da ChIP-seq experiments in the adult fruit fly heads showed widespread Da binding to promoter regions, suggesting the importance of Da in the adult *Drosophila*. Given the abundance of E-box motifs in the genome, it is challenging to determine which genes are affected by a specific Da binding site and which processes are regulated by Da in the adult nervous system using only binding-based analysis.

10.1523/ENEURO.0051-25.2025.f1-1 Figure 1-1 **FLAG-Da ChIP-seq results.** Peaks that were significantly enriched in 3xFLAG-Da samples compared to white* samples are listed. Genomic location, annotation, gene related to the location, normalized counts, enrichment fold, FDR and p value are shown for each enriched peak. Download Figure 1-1, XLS file.

### Extramacrochaete inhibits the transcriptional activity of Daughterless in *Drosophila* neurons

**Figure 2.**
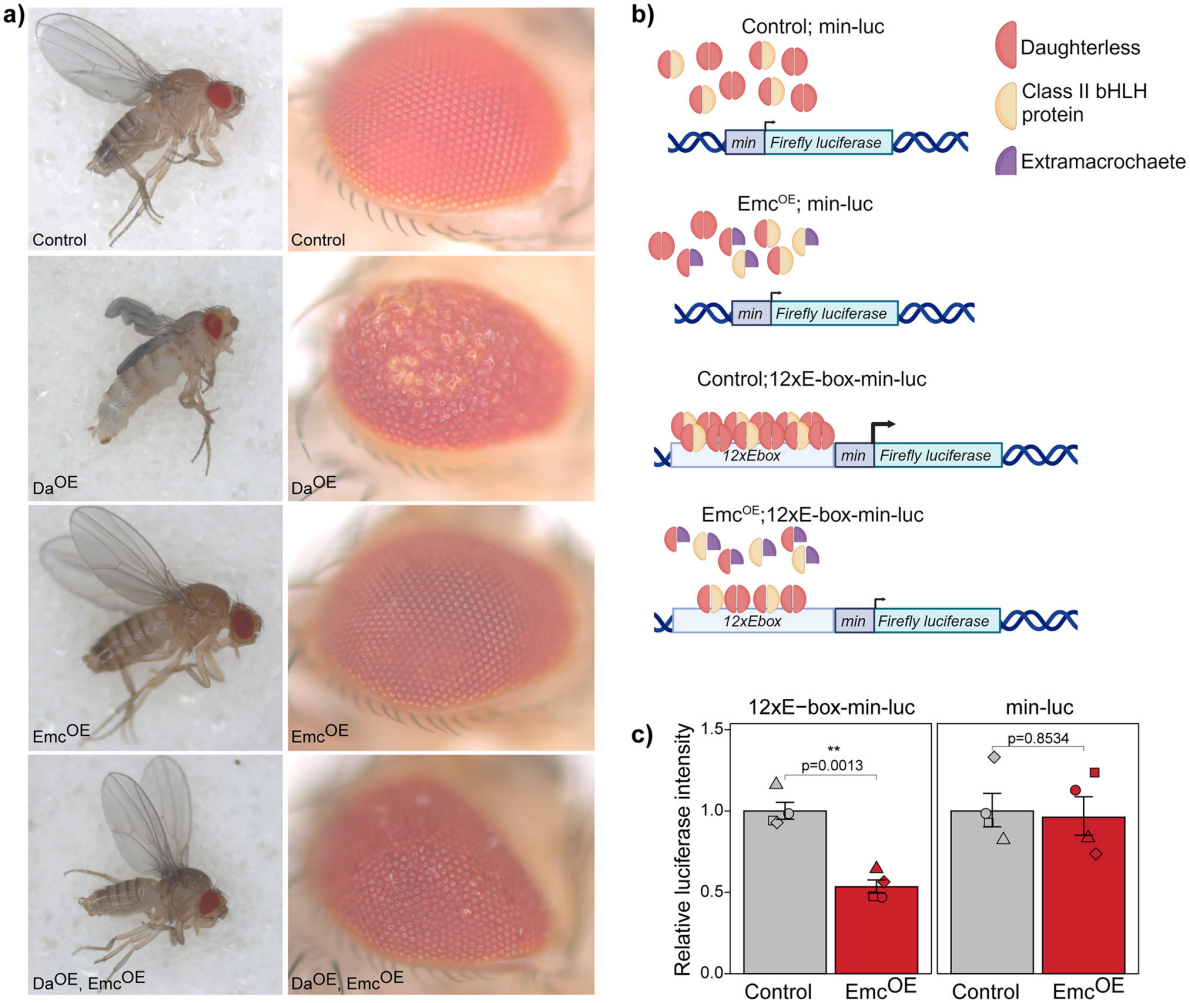
Emc overexpression impairs Da transcriptional activity in vivo. ***a***, Light micrographs of adult flies; control, elavC155-Gal4/white*; Da^OE^, elavC155-Gal4 > *da*; Emc^OE^, elavC155-Gal4 > emc; Da^OE^, Emc^OE^, elavC155-Gal4 > Da,Emc. ***b***, Schematic representation of in vivo luciferase reporter assay (created using BioRender.com). Transgenic flies containing minimal promoter (min) and a firefly luciferase reporter gene (*Firefly luciferase)* for min-luc or *12xCAGCTG* E-boxes (12xE-box), minimal promoter, and a firefly luciferase reporter gene for 12xE-box-min-luc were used. Emc was overexpressed (Emc^OE^) using elavC155-Gal4 driver. As a control, elavC155-Gal4 and reporter construct containing flies were crossed to white* flies (control; min-luc and control;12xE-box-min-luc). ***c***, Results of the luciferase reporter assay. Luciferase activities were measured and shown as fold change compared with the control, the replicates are shown as individual shapes, and error bars indicate standard error of the mean (SEM); *n* = 4, two-tailed Student’s paired *t* test.

To identify the genes regulated by Da in the adult *Drosophila* nervous system, we required a model where Da function is inhibited. Our previous findings indicated that heterozygous *da* null mutation did not affect larval memory (Tamberg et al., 2020) potentially due to autoregulation controlling Da protein levels (Smith and Cronmiller, 2001; Bhattacharya and Baker, 2011). Therefore, we opted to utilize neuron-specific overexpression of Emc, a known negative regulator of Da function (Ellis et al., 1990; Van Doren et al., 1991; Cabrera et al., 1994; Bhattacharya and Baker, 2011). To confirm Da inhibition by Emc, we performed experiments where Da, Emc, or both were overexpressed in neurons using a pan-neuronal driver *elavC155-Gal4* (Fig. 2*a*). Overexpression of Da alone resulted in severe wing and eye phenotypes in adult flies and negatively affected viability, with only a small fraction of larvae reaching pupation, resulting in no males and only a few females emerging from the pupae (Fig. 2*a*). Simultaneous overexpression of Emc mitigated these phenotypes, demonstrating that Emc counteracts the detrimental effects of Da overexpression (Fig. 2*a*). Notably, overexpression of Emc alone did not produce any visible effects on wing or eye development (Fig. 2*a*).

We further investigated the effects of Emc overexpression on endogenous Da activity using in vivo luciferase reporter assay in adult *Drosophila* brains. For that we generated transgenic flies where *Firefly luciferase* coding region is under the control of *12xE-box* motifs and a *Drosophila melanogaster* minimal promoter or only minimal promoter as a control. The *12xCAGCTG* E-boxes were used since based on our ChIP-seq data, this is the most prevalent E-box bound by Da in adult fly heads (Fig. 1*d*,*e*). In these flies, we employed neuron-specific overexpression of Emc by *elavC155-Gal4* to inhibit the transcriptional activity of endogenous Da (Fig. 2*b*). In luciferase reporter assay, E-box–driven luciferase expression measured by its activity was indeed significantly decreased when Emc was overexpressed in neurons (Fig. 2*c*). Emc overexpression had no effect on the reporter lacking the E-boxes (Fig. 2*c*). These results validated the use of Emc overexpression in neurons to study Da downstream genes and its functions in the adult brain.

### Inhibition of Daughterless activity affects genes involved in behavior, memory, synaptic signaling, translation, and metabolic processes

**Figure 3.**
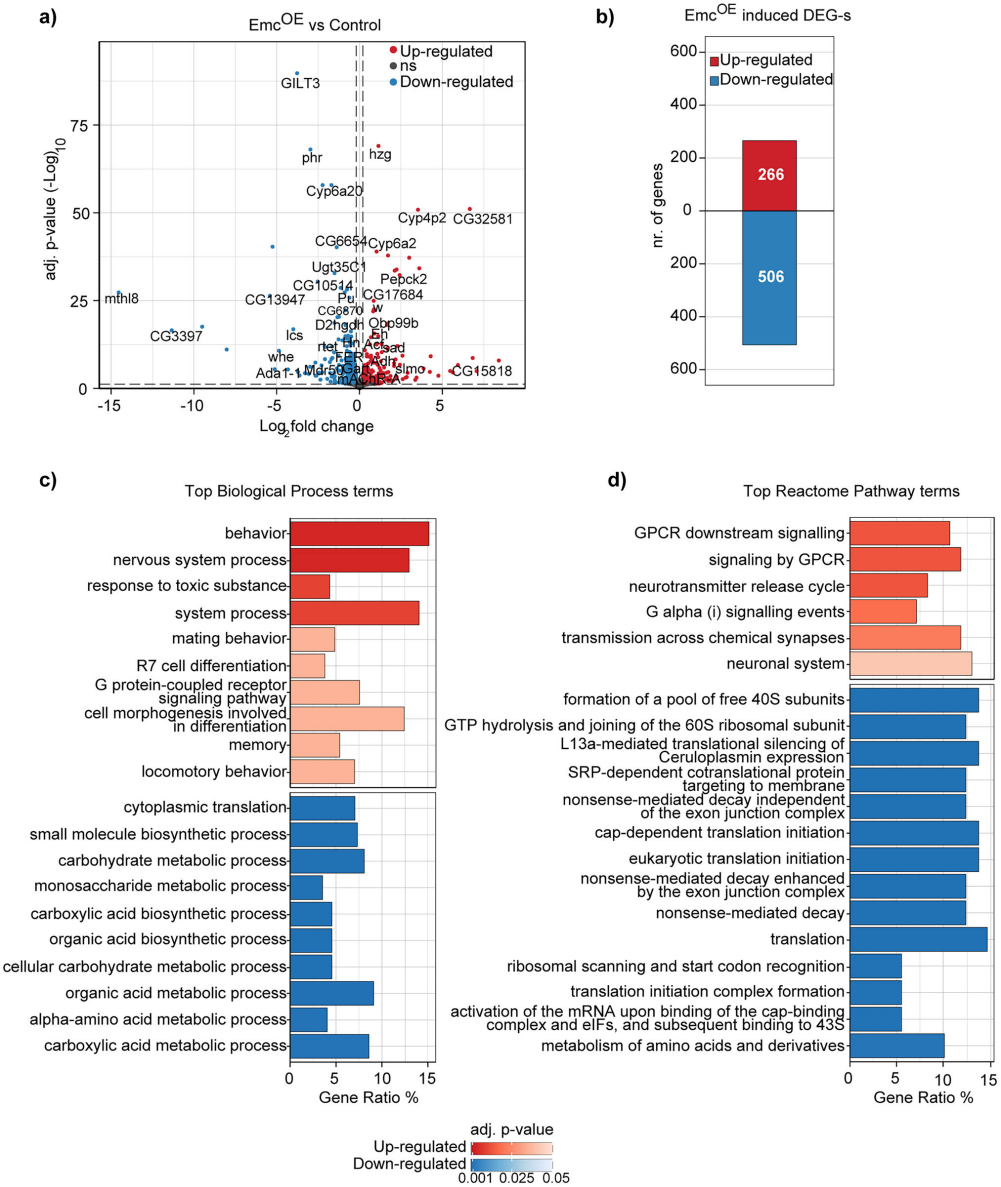
Widespread transcriptional changes after pan-neuronal Da inhibition by Emc. ***a***, Differentially expressed genes (DEGs) in the brains of Emc^OE^ (elavC155-Gal4 > emc) flies compared with the control (elavC155-Gal4xwhite*). On *x*-axis, fold changes are shown in log_2_ scale, and on *y*-axis adjusted *p* values (BH normalization, DESeq2) are shown in −log_10_ scale. Dotted lines represent cutoff values for ±0.2 log_2_ fold change and 0.05 adj. *p* value. Significantly upregulated and downregulated genes are listed in Extended Data Figure 3-1. Levels of *emc*, *da*, and Class 2 bHLH protein mRNAs are shown in Extended Data Figure 3-2. ***b***, Summary of DEGs from the RNA-seq experiment, above and below zero, indicates up- and downregulated genes, respectively. ***c***, GO terms and (***d***) Reactome Pathway defined pathways dysregulated by Emc overexpression; color gradient represents adjusted *p* values; red and blue indicate up- and downregulated gene cohorts, respectively. Biological processes affected by Da inhibition are shown in Extended Data Figure 3-3 and Reactome Pathways in Extended Data Figure 3-4.

To study the roles of Da in adult *Drosophila* nervous system, we investigated the brain transcriptome of flies where Da activity was suppressed by pan-neuronal Emc overexpression. In Emc^OE^ brains, 266 genes were significantly upregulated, and 506 genes were downregulated compared with control brains (Fig. 3*a*,*b*; Extended Data Fig. 3-1). *emc* was successfully overexpressed, and this had no effect on the levels of *da* (Extended Data Fig. 3-2). Additionally, our RNA-seq analysis showed that 9 out of 23 Class 2 bHLH protein genes are expressed at moderate levels in the adult *Drosophila* brains—*Fer1*, *Fer2*, *oli*, *net*, *tx*, *sage*, *dimm*, *HLH3B*, and *HLH4C* (Massari and Murre, 2000; Ledent and Vervoort, 2001; Extended Data Fig. 3-2). This provides the possibility for Da to form heterodimers to regulate transcription. In our transcriptomics experiments, in addition to inhibiting Da directly, overexpressed Emc could form non-DNA binding dimers with neuronally expressed Class 2 bHLH proteins, thereby affecting the expression of Da homodimer and Da—Class 2 heterodimer target genes.

10.1523/ENEURO.0051-25.2025.f3-1 Figure 3-1 **Differential gene expression caused by Da inhibition by Emc overexpression.** Significantly up-regulated and down-regulated genes are listed. Gene name, average counts and counts in each genotype, log2 fold change and adjusted p values are shown. Download Figure 3-1, XLS file.

10.1523/ENEURO.0051-25.2025.f3-2 Figure 3-2 **Expression of Da and its dimerization partners in the adult brains when Emc is overexpressed.** Normalized counts of *emc, da, l(1)sc, sc, ac, ase, cato, ato, amos, tap, twi, nau, HLH54F, hand, CG33557, Fer1, Fer2, Fer3, oli, net, tx, sage, dim, HLH3B and HLH4C* are shown from Emc overexpression RNA-seq experiments. Classification is based on Ledent and Vervoort, 2001, and Massari and Murre, 2000. Emc^OE^ – elavC155-Gal4 > emc, Control – elavC155-Gal4xwhite*. The replicates are shown as individual shapes and error bars represent standard error of the mean (SEM)**.** ***p < 0.001; ns, not significant; Benjamini-Hochberg procedure (BH) -adjusted p-values. Download Figure 3-2, TIF file.

10.1523/ENEURO.0051-25.2025.f3-3 Figure 3-3 **Biological processes affected by Da inhibition by Emc overexpression.** Gene ontology analysis was conducted on differentially expressed genes caused by Emc overexpression and biological processes affected are listed. Description of the categories, genes ID-s of differentially expressed genes belonging to the categories, p values, adjusted p values and q values are shown. Download Figure 3-3, XLS file.

**Figure 4.**
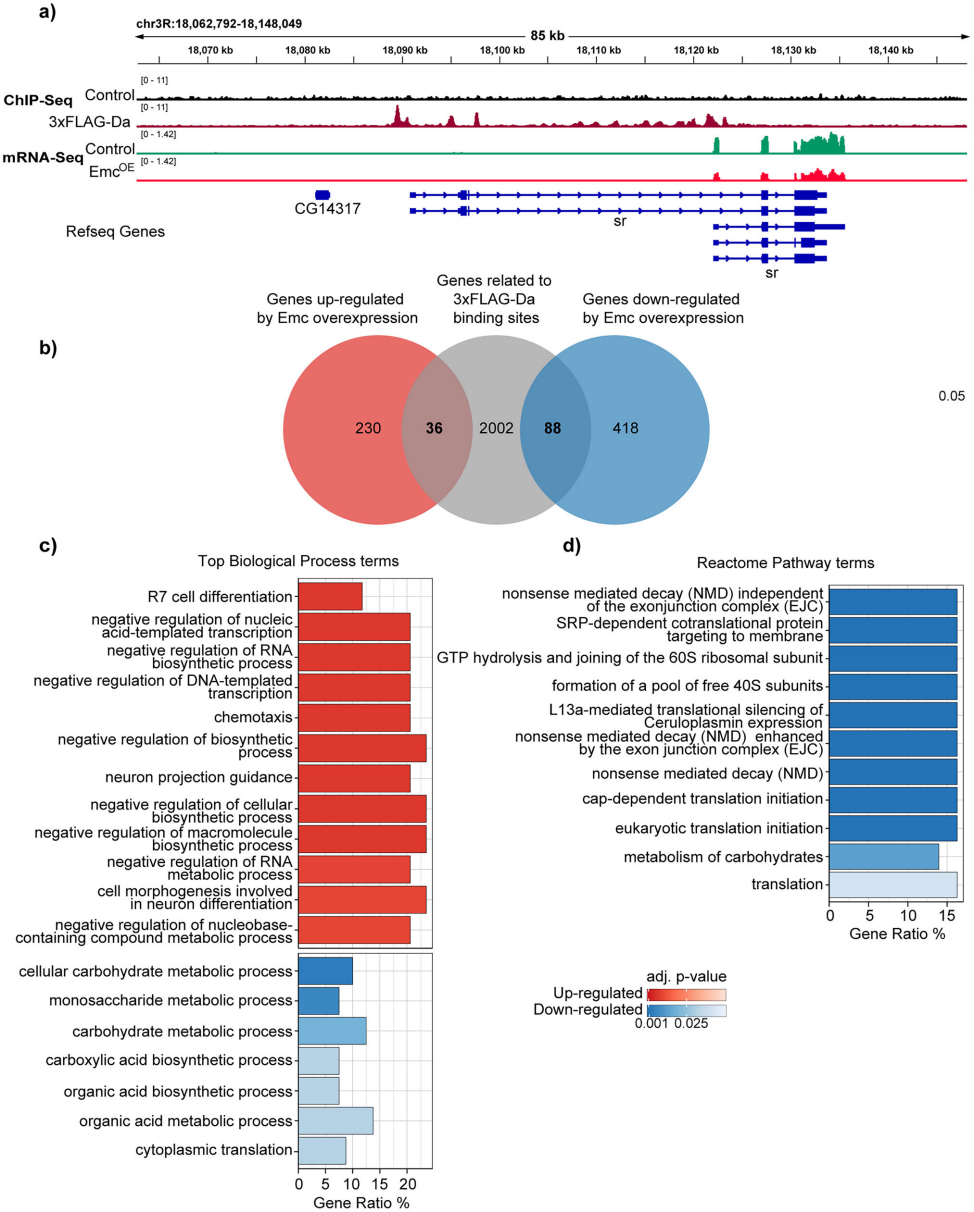
Pathways directly regulated by Da. ***a***, Visualization of anti-FLAG ChIP-seq data from adult *Drosophila* heads of 3xFLAG-Da and control flies and RNA-seq data from adult brains of Emc^OE^ (elavC155-Gal4 > emc) flies compared with control (elavC155-Gal4xwhite*) flies. ChIP-Seq data were visualized as fold enrichment over input and RNA-Seq data as counts per million aligned reads. Biological replicates were merged for visualization, with *n* = 2 for ChIP-Seq and *n* = 4 for RNA-Seq. As an example, *stripe (sr)* gene locus is shown, where 3xFLAG-Da binds to the promoter regions, and expression in RNA-Seq is lower in Emc^OE^. ***b***, Venn diagram of common statistically significant genes from 3xFLAG-Da ChIP-seq and Emc^OE^ RNA-seq datasets. For 3xFLAG-Da ChIP-seq, statistically significant genes were defined as log_2_ fold change > 1 (compared with white*) and false discovery rate ≤ 0.05. For Emc^OE^ RNA-seq, statistically significant genes were determined as follows: counts ≥20 at least in control or Emc^OE^ samples, log_2_ fold change >0.2 or <−0.2 and *p*-adjusted values ≤ 0.05. Genes that were upregulated or downregulated by Emc overexpression and that contained FLAG-Da ChIP peaks are listed in Extended Data Figure 4-1. ***c***, GO terms and (***d***) Reactome Pathway terms for common genes. Color gradient represents adjusted *p* values (BH); red and blue indicate up- and downregulated gene cohorts in the RNA-seq data, respectively. Biological processes directly regulated by Da are shown in Extended Data Figure 4-2 and Reactome Pathways in Extended Data Figure 4-3.

10.1523/ENEURO.0051-25.2025.f3-4 Figure 3-4 **Reactome pathways affected by Da inhibition by Emc overexpression.** Gene ontology analysis was conducted on differentially expressed genes caused by Emc overexpression and Reactome pathways affected are listed. Description of the categories, genes ID-s of differentially expressed genes belonging to the categories, p values, adjusted p values and q values are shown. Download Figure 3-4, XLS file.

To explore the processes regulated by Da, we performed GO enrichment analysis on the differentially expressed gene data. Notably, 15% of all upregulated genes were associated with behavior (Fig. 3*c*; Extended Data Fig. 3-3). The term “behavior” included genes that were also enriched in memory, learning and cognition, locomotion, mating, reproduction, and male courtship behavior (Extended Data Fig. 3-3). Inhibition of Da activity also resulted in upregulation of genes associated with photoreceptor R7 differentiation, G-protein–coupled receptor signaling, cell projection morphogenesis, regulation of neurotransmitter, and synapses (Fig. 3*c*; Extended Data Fig. 3-3). For example, among the upregulated genes were the following synapse associated genes: *5-HT1A* (serotonin receptor; Gasque et al., 2013), *Tbh* (key-limiting enzyme in octopamine synthesis; Brembs et al., 2007), *SLC22A* (acetylcholine uptake; Gai et al., 2016), *brp* (synaptic vesicle release; Hallermann et al., 2010), *Syx1A* (neurotransmitter release; Schulze et al., 1995), *unc-13* (synaptic vesicle exocytosis; Aravamudan et al., 1999), *VGAT* (GABA packaging into synaptic vesicles; Fei et al., 2010), *Syt7* (synaptic vesicle exocytosis; Guan et al., 2020), and *Vmat* (dopamine, serotonin, and octopamine packaging into secretory vesicles; Greer et al., 2005; Extended Data Fig. 3-3). Downregulated genes were generally associated with pathways involved in translation and metabolism (Fig. 3*c*; Extended Data Fig. 3-3). To further elucidate pathways that are regulated by Da, we utilized a manually curated and peer-reviewed Reactome pathway database. Similar to GO analysis, genes associated with G-protein–coupled receptor signaling, neurotransmitters, and synapses were upregulated when Da was inhibited (Fig. 3*d*; Extended Data Fig. 3-4). Additionally, downregulated genes were associated with translation and metabolism but also with the nonsense-mediated decay pathway (Fig. 3*d*; Extended Data Fig. 3-4). In conclusion, our RNA-seq experiments of adult *Drosophila* brains, where Da function was inhibited by Emc overexpression, demonstrated that Da regulates genes related to metabolism, translation, and behavior including memory, neurotransmitter transport and release, and synapses.

### Daughterless directly regulates genes associated with neuronal projection guidance, metabolism, and translation in the adult *Drosophila* brains

Next, to further elucidate the roles of Da in the adult nervous system, we were interested which genes and processes are directly regulated by Da. For that, we performed integrated analysis of the two generated datasets—differentially expressed genes in the brain where Da function was inhibited by Emc overexpression and 3xFLAG-Da binding data from ChIP-seq experiment (Fig. 4*a*). Overlap between the datasets show that 3xFLAG-Da bound 36 of the upregulated genes (13.5%, two-sided Fisher's exact test, Holm adjusted *p* value 0.0372) and 88 of downregulated genes (17.4%, two-sided Fisher's exact test, Holm adjusted *p* value 2.04 × 10^−8^; Fig. 4*b*; Extended Data Fig. 4-1), consistent with the notion that Da functions as activator of transcription. To investigate the functions of direct Da target genes, we used enrichment analysis. GO analysis revealed that 20% of the upregulated genes were associated with regulation of neuron projection guidance (Fig. 4*c*; Extended Data Fig. 4-2). This included the following genes involved in axon guidance: *SoxN* (HMG-domain transcription factor; Girard et al., 2006), *LRP1* (LDL receptor protein; Li et al., 2020), *sbb* (transcriptional coregulator; Kaminker et al., 2002), *Dab* (adaptor protein; Song et al., 2010), *side* (transmembrane protein; Siebert et al., 2009), and *RhoGAP100F* (Rho GTPase-activating protein; Holbrook et al., 2012; Extended Data Fig. 4-2). The downregulated genes were enriched in terms associated with metabolism and translation (Fig. 4*c*; Extended Data Fig. 4-2). In addition, we used the Reactome pathway analysis of the Da direct target genes. Genes directly regulated by Da were involved in translation and nonsense-mediated decay (Fig. 4*d*; Extended Data Fig. 4-3). Also, carbohydrate metabolism-related genes were affected. In conclusion, by combining 3xFLAG-Da binding and Emc overexpression transcriptomics datasets, we showed that Da directly regulates genes that are associated with neuronal projection guidance, metabolism, and translation.

10.1523/ENEURO.0051-25.2025.f4-1 Figure 4-1 **Da target genes.** Genes that were up-regulated or down-regulated by Emc overexpression and that contained FLAG-Da ChIP peaks are listed. Gene name, average counts and counts in each genotype, log2 fold change and adjusted p values are shown. Additionally, peaks that were significantly enriched in 3xFLAG-Da samples compared to white* samples and are related to genes that were differentially expressed by Emc overexpression are listed. Genomic location, annotation, gene related to the location, normalized counts, enrichment fold, FDR and p value are shown for each enriched peak. Download Figure 4-1, XLS file.

10.1523/ENEURO.0051-25.2025.f4-2 Figure 4-2 **Biological processes regulated by Da target genes.** Gene ontology analysis was conducted on the Da target gene data set and biological processes affected are listed. Description of the categories, genes ID-s of differentially expressed genes belonging to the categories, p values, adjusted p values and q values are shown. Download Figure 4-2, XLS file.

10.1523/ENEURO.0051-25.2025.f4-3 Figure 4-3 **Reactome pathways regulated by Da target genes.** Gene ontology analysis was conducted on the Da target gene data set and Reactome pathways affected are listed. Description of the categories, genes ID-s of differentially expressed genes belonging to the categories, p values, adjusted p values and q values are shown. Download Figure 4-3, XLS file.

### Daughterless is important for learning of adult *Drosophila*

**Figure 5.**
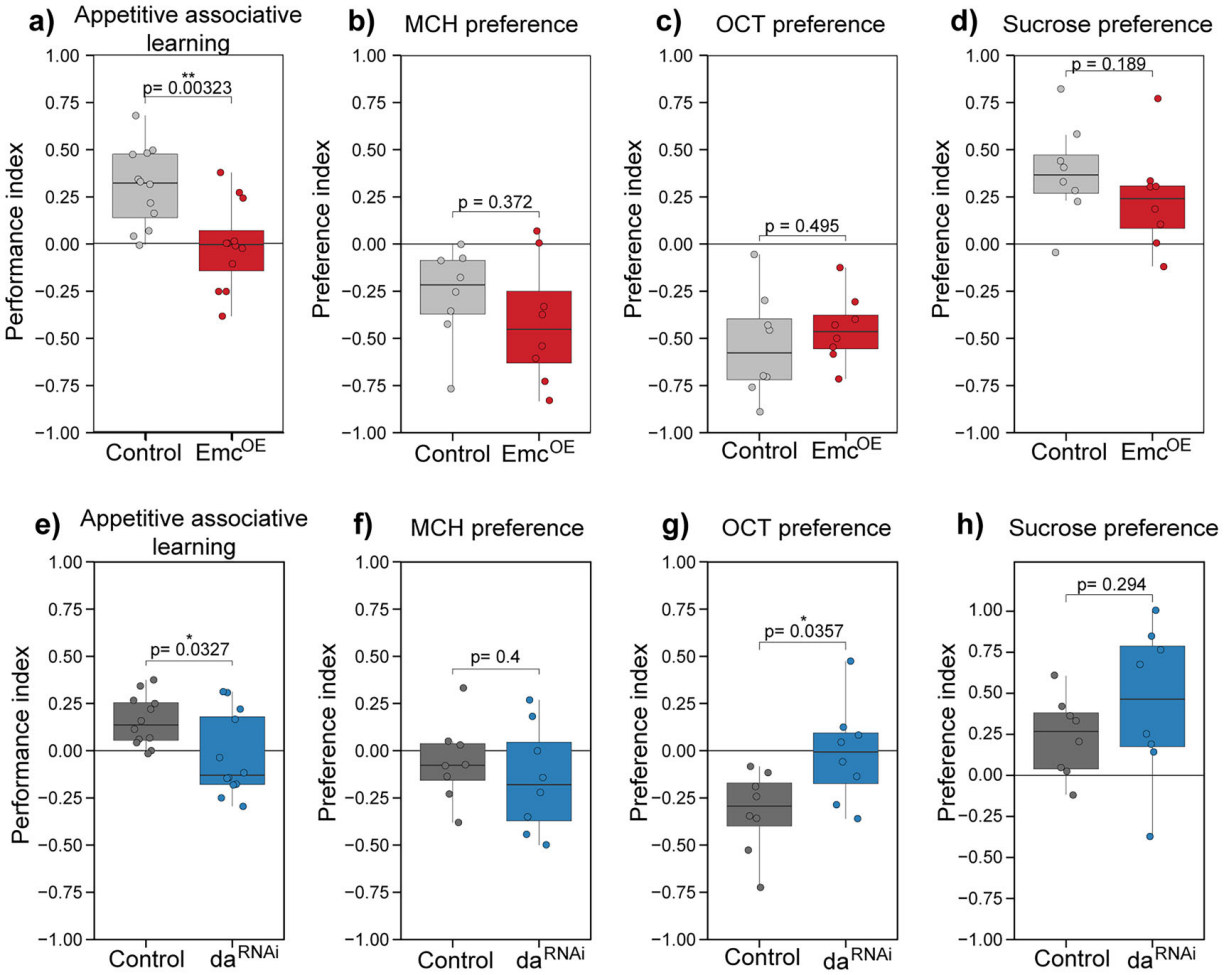
Appetitive associative learning is impaired in adult *Drosophila* with pan-neuronal Da inhibition by Emc. Performance index of 1-d-old Emc^OE^ (elavC155-Gal4 > emc) flies is significantly reduced compared with control (elavC155-Gal4xwhite*) flies (***a***), and performance index of da^RNAi^ (nSyb-Gal4 > da^RNAi^) flies is significantly reduced compared with control (nSyb-Gal4xwhite*) flies (***e***). Preference index toward MCH is shown on ***b*** and ***f***, OCT (3-octanol) on ***c*** and ***g***, and sucrose on ***d*** and ***h***. Performance and preference indexes are visualized using box-whisker plots that show the median, the 25% and 75% quartiles (hinges); the upper whisker extends from the hinge to the largest value no further than 1.5 of the interquartile range from the hinge; the lower whisker extends from the hinge to the smallest value at most 1.5 * interquartile range of the hinge, individual data points are presented as small dots; *n* = 12 for ***a*** and ***e***; *n* = 8 for ***b***, ***c***, ***d***, ***f***, ***g***, and ***h***. *p* values were calculated using two-sided Wilcoxon rank-sum test. Expression patterns of pan-neuronal Gal4 drivers used are shown in Extended Data Figure 5-1.

The RNA-seq experiment showed that in adult *Drosophila* brains, Da regulates genes associated with learning, memory, and synaptic signaling. In addition, according to our experiments, genes associated with neuronal projection guidance are direct targets of Da in the adult brain. Next, to validate Da involvement in memory formation of adult *Drosophila*, we carried out appetitive associative learning experiments using neuronal Emc overexpression flies. The median performance index of Emc^OE^ flies was close to 0, and the median performance index of the control flies was ∼0.3 (Fig. 5*a*), indicating a severe memory impairment of the Emc^OE^ flies. Our experiments demonstrated that inhibiting Da activity through Emc overexpression significantly reduced the learning ability of the flies. We performed additional learning experiments with flies where *da* was silenced using RNAi approach to further validate that Da itself is involved in memory formation. Because silencing *da* by *elavC155-Gal4* driver was lethal at pupal stage, we used another pan-neuronal driver—*nSyb-Gal4*. Memory of the *da^RNAi^* flies was significantly impaired compared with control flies (Fig. 5*e*) demonstrating again the importance of Da in memory formation of adult *Drosophila*.

10.1523/ENEURO.0051-25.2025.f5-1 Figure 5-1 **Expression of 3xFLAG-Da, *elavC155-Gal4* and *nSyb-Gal4* in the adult *Drosophila* brain. (**a) and (g) show 3xFLAG-Da expression in the dorsal part of the brain, (d) and (j) show 3xFLAG-Da expression in the ventral part of the brain in magenta; nls-GFP expression shows the expression pattern of the drivers in green – (b) – *elavC155-Gal4* dorsal part of the brain, (e) – *elavC155-Gal4* ventral part of the brain, (h) – *nSyb-Gal4* dorsal part of the brain, (k) - *nSyb-Gal4* ventral part of the brain; on (c), (f), (i) and (l) 3xFLAG-Da and the driver’s signals are merged. White arrows point to some co-expression. Download Figure 5-1, TIF file.

To test if these results were truly caused by memory impairment and not by changes in the sensing of smell and taste of the flies, we carried out preference experiments toward sucrose and the odors. Both of the odors—3-octanol (OCT) and 4-methyl cyclohexanol (MCH)—were aversive to the Emc^OE^ and control flies as shown before (Hussain et al., 2018; Fig. 5*b*,*c*). Although we noticed a small reduction of sucrose preference in Emc^OE^ flies (median preference of the control flies was ∼0.35 and the Emc^OE^ flies 0.25), this difference was not statistically significant and both flies preferred sucrose solution to water (Fig. 5*d*). *da^RNAi^* flies were able to sense MCH and sucrose but failed to sense OCT (Fig. 5*f*–*h*). This is probably caused by different driver lines used for Emc^OE^ and *da^RNAi^*. *elavC155-Gal4* is expressed strongly in the mushroom bodies, but the expression is weaker elsewhere, and *nSyb-Gal4* is expressed more widely (Extended Data Fig. 5-1).

**Figure 6.**
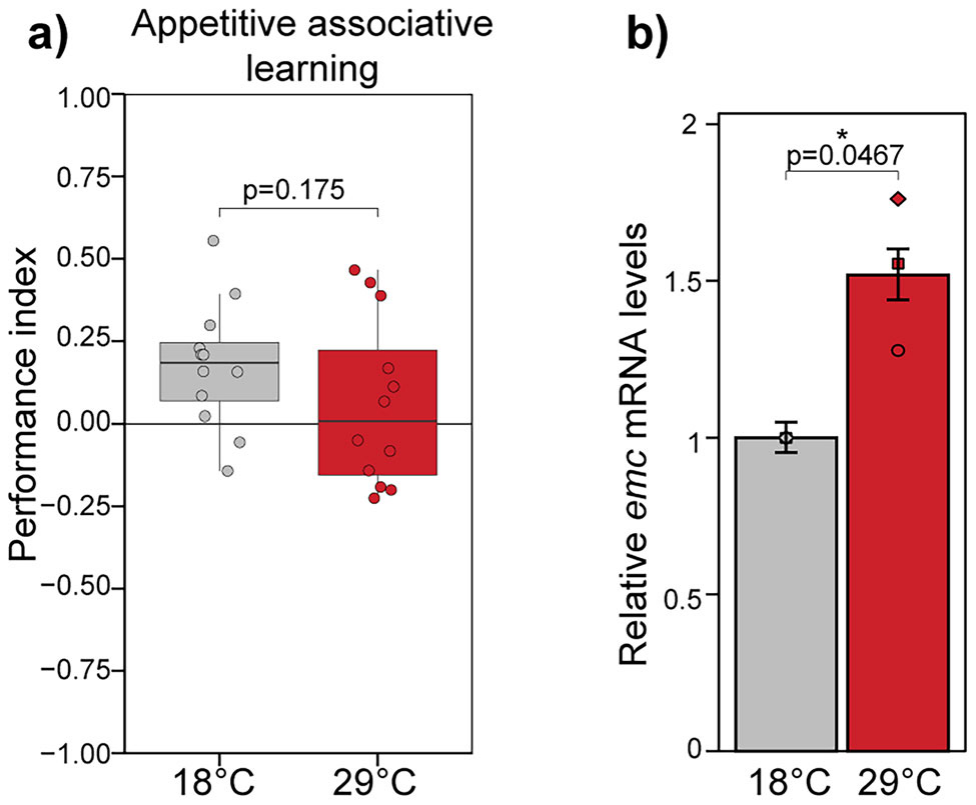
Inhibiting Da in the adult brains after pupariation does not significantly effect memory of the flies. ***a***, Performance index of *ts-Gal80*;*elavC155-Gal4* *>* *emc* flies starved in 29°C for 24 h is not significantly changed compared with flies starved in 18°C. Performance indexes are visualized using box-whisker plots that show the median, the 25% and 75% quartiles (hinges); the upper whisker extends from the hinge to the largest value no further than 1.5 of the interquartile range from the hinge; the lower whisker extends from the hinge to the smallest value at most 1.5 * interquartile range of the hinge; individual data points are presented as small dots; *n* = 12 *p* value was calculated using two-sided Wilcoxon rank-sum test. ***b***, qPCR results of cDNA from *ts-Gal80*;*elavC155-Gal4* *>* *emc* flies kept in 29 or 18°C for 24 h. Relative *emc* mRNA levels were calculated and shown in fold change compared with flies in 18°C. Replicates are shown as individual shapes, and error bars indicate SEM; *n* = 3, two-tailed Student’s paired *t* test.

Next, to further investigate involvement of Da in learning of adult flies, we were interested if memory impairment is caused by inhibiting Da activity during development or in the adult brain. *elavC155-Gal4* is expressed from embryonic stages, and the memory impairment caused by Emc overexpression using this driver could be developmental. We decided to use temperature-sensitive *tubulin-Gal80 (ts-Gal80)* transgene to activate Emc overexpression in the adult flies after pupariation. Gal80 inhibits Gal4, and the ts-Gal80 is active in 18°C and degrades in 29°C. *ts-Gal80;elavC155* *>* *emc* flies were grown in 18°C and transferred to 29°C after eclosion from the pupae where they were starved for 24 h for memory experiments. Control flies with the same genotype were starved in 18°C for 24 h. Although *emc* was successfully overexpressed in the brains of the flies who were transferred to 29°C, their learning was not significantly impaired (Fig. 6*a*,*b*). This shows that inhibiting Da activity in the adult brains using Emc overexpression is not sufficient to cause memory impairment although Da regulates genes associated with memory in the adult brains. Collectively, our results show that Da is required for memory of adult *Drosophila* acting already during development.

### Overexpression of Daughterless causes reduction of the translation rate in adult *Drosophila* brains

**Figure 7.**
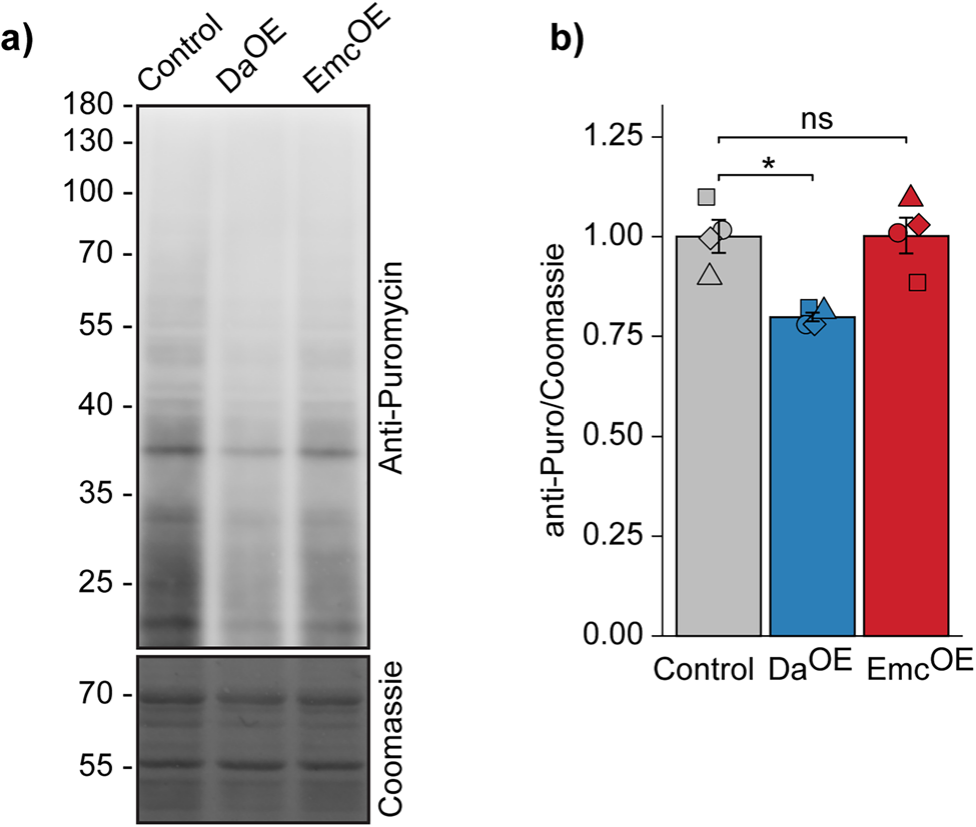
Effects of neuronal Emc overexpression and Da overexpression on translation rates in adult *Drosophila* brain. ***a***, Representative Western blot experiment of brains of 3–4-h-old flies treated with 5 µM puromycin for 1 h to label de novo synthesized proteins and detected using Western blot analysis (Anti-Puromycin); Coomassie staining was used for normalization; numbers on the left represent molecular weight in kilodalton; control, elavC155-Gal4xwhite*; Da^OE^, elavC155-Gal4 > Da; Emc^OE^, elavC155-Gal4 > Emc. ***b***, Results of densitometric analysis of Western blot. The puromycin signals were normalized using Coomassie signals. The mean results from four independent experiments are shown. Results are presented as relative fold change against control, the replicates are shown as individual shapes, and error bars indicate SEM; *p < 0.05; ns, not significant; two-tailed Student's paired *t* test.

The FLAG-Da ChIP-seq and Emc^OE^ transcriptomics experiments showed that Da directly regulates a number of ribosome protein genes (Extended Data Fig. 4-1). To further investigate the role of Da in regulating protein synthesis, we used ex vivo labeling of de novo synthesized proteins using puromycin (Villalobos-Cantor et al., 2023). Puromycin incorporates into newly synthesized polypeptide chains and allows detection using anti-puromycin antibodies. We tested different times of labeling and observed that 60 min gives strongest labeling without apparent shift toward lower molecular weight (degraded or truncated) proteins (data not shown). Therefore 60 min of labeling was used in all subsequent experiments. In addition to flies overexpressing Emc, we included flies with pan-neuronal Da overexpression to explore potential opposing effects on translation rates. Western blot experiments with anti-puromycin antibodies showed no effect of Emc overexpression on protein synthesis. In contrast, a 20% decrease in general translation rate was observed in Da overexpressing fly brains (Fig. 7*a,b*). Inhibiting Da activity by Emc overexpression using *elavC155-Gal4* is possibly not enough to cause change in global translation rate although it leads to transcriptional dysregulation of ribosomal protein genes in the adult *Drosophila* brains.

## Discussion

Da is well known for its functions during the development of *Drosophila*, and it is crucial for the development of the nervous system (Caudy et al., 1988; Tepass and Hartenstein, 1995; Wong et al., 2008; Bhattacharya and Baker, 2011; Tamberg et al., 2015; Wang and Baker, 2015; Li and Baker, 2019). In recent years, evidence has emerged that Da also plays a role in the adult nervous system (D’Rozario et al., 2016; Tamberg et al., 2020). Moreover, we have previously shown that Da is expressed in the adult *Drosophila* brain (Tamberg et al., 2020). Here, we set out to elucidate the roles of Da in the adult nervous system.

To investigate Da transcriptional activity, we generated a reporter fly line where expression of *Firefly luciferase* gene is under the control of *CAGCTG* E-boxes, since this E-box sequence was preferred by FLAG-Da in ChIP-seq experiments. Using this reporter fly line, we validated that Emc acts as a repressor of Da activity in the adult *Drosophila* brains. Previous work on different E-box binding preferences of Da-Da homodimers or Da-class II bHLH heterodimers have shown that both homodimers and heterodimers bind to *CAGCTG* E-box sequence (Cabrera and Alonso, 1991; Jarman et al., 1993; Kunisch et al., 1994). In addition, Da-Da homodimers have been shown to bind strongly to *CATTTG*, *CATCTG*, and *CACCTG* (Kunisch et al., 1994) and weakly to *CAGGTG* (Jarman et al., 1993) E-boxes. Strong binding of the heterodimers has been reported to *CAGCTG*, *CAGGTG*, and *CACGTG* E-box sequences (Cabrera and Alonso, 1991; Jarman et al., 1993; Kunisch et al., 1994). In addition, flanking regions of the E-boxes also influence the binding of different Da-proneural protein complexes (Powell et al., 2004).

To investigate processes regulated by Da in the adult *Drosophila* brain, we analyzed transcriptome of flies where Da function was inhibited by neuron-specific overexpression of the negative regulator of Da—Emc (Ellis et al., 1990; Van Doren et al., 1991; Cabrera et al., 1994; Bhattacharya and Baker, 2011; Waddell et al., 2019). In addition to forming dimers with Da, Emc also dimerizes with Class 2 bHLH proteins and inhibits their dimerization capability with Da (Ellis et al., 1990; Cabrera et al., 1994). Our transcriptomics results show that there are several Class 2 bHLH protein genes expressed in the adult *Drosophila* brains. There is evidence of some vertebrate Class 2 bHLH proteins that regulate transcription without needing to dimerize with E-proteins (Torres-Machorro, 2021); however, there is no evidence that this happens in *Drosophila* with neuronally expressed Class 2 bHLH proteins. Homodimerization has only been shown for Twist which is not expressed in the adult brain according to our RNA-seq data (Castanon et al., 2001). This means that overexpressed Emc inhibits Da from homodimerizing and heterodimerizing with Class 2 bHLH proteins and thereby affecting its target genes in the adult *Drosophila* brains.

Our transcriptomics analysis showed that in the adult *Drosophila* brains, Da regulates synapses, memory, metabolism, and translation. The regulation of synaptic proteins in the adult brain is in agreement with our and others’ previous results from larval brain, where Da has been shown to regulate the expression of synaptic proteins Synapsin, Discs large 1, and Neurexin (D’Rozario et al., 2016; Tamberg et al., 2020). Da ortholog TCF4 also regulates memory and synaptic transmission in mouse models and also genes regulating these processes in human cell models (D’Rozario et al., 2016; Li et al., 2019; Sarkar et al., 2021; Davis et al., 2024). Furthermore, to elucidate the roles of Da in adult nervous system, we performed ChIP-seq experiments to investigate which genes are directly regulated by Da. Da binding-site data from adult *Drosophila* heads together with Emc^OE^ transcriptomics data from the brains revealed that genes associated with development of neuronal projections, metabolism, and translation are direct targets of Da. Our discovery that Da regulates genes involved in metabolism and translation is novel opens the possibility for investigating human E-proteins, including TCF4, also in these contexts.

We further confirmed the involvement of Da in the memory formation of adult *Drosophila* using behavioral experiments. Flies with inhibited Da activity displayed no memory, while control flies were capable of learning. These experiments together with our previous results showing that silencing of *da* in the larval brain impairs the appetitive associative learning (Tamberg et al., 2020) support the transcriptomics data that Da regulates genes that function in learning of the fruit fly.

Our findings showing that Da directly regulates genes involved in translation in the adult brain are novel and expand the knowledge about the functions of E-proteins. Current understanding is that memory is based on synaptic plasticity and changes in synapses need protein synthesis (Sossin and Costa-Mattioli, 2019; Bin Ibrahim et al., 2024; Di Liegro et al., 2024). This means that the processes regulated by Da in adult *Drosophila* brains—synaptic transmission, neuronal projection morphogenesis, and memory—could be linked through Da-regulated general translation. Moreover, it has been shown that translation is often dysregulated in autism spectrum disorders (Sossin and Costa-Mattioli, 2019; Longo and Klann, 2021). Mutations in Da human ortholog TCF4 cause a severe autism spectrum disorder PTHS (Zollino et al., 2019). *Drosophila*'s Da can be further investigated in the perspective of protein synthesis, and this could give new insights into the mechanisms of PTHS. Moreover, this opens a new avenue to possibly relieve some of the symptoms of PTHS. For example, it has been shown that drugs that normalize translation rates have positive effects on the autistic behaviors in fragile X syndrome mouse models (Gkogkas et al., 2014; Gantois et al., 2017).

In conclusion, we have elucidated the roles of Da in the adult *Drosophila* nervous system showing that Da regulates genes involved in synaptic transmission and memory, and genes associated with metabolism and translation are direct targets of Da. Understanding the roles of Da in adult brain possibly give insights about the roles of TCF4 in the adult brain that could be beneficial in understanding the mechanisms of the neurological diseases associated with *TCF4*. Moreover, our results indicate that Da and possibly TCF4 are involved in regulation of translation. This new avenue could be useful for developing therapies to alleviate the symptoms of PTHS.

## Data Availability

Raw sequencing data have been deposited in the Gene Expression Omnibus database under the following accession codes: GSE279158 (RNA-seq) and GSE279107 (ChIP-seq).

## Synthesis

Reviewing Editor: Fabienne Poulain, University of South Carolina Decisions are customarily a result of the Reviewing Editor and the peer reviewers coming together and discussing their recommendations until a consensus is reached. When revisions are invited, a fact-based synthesis statement explaining their decision and outlining what is needed to prepare a revision will be listed below. The following reviewer(s) agreed to reveal their identity: NONE.

Although Daughterless/TCF4 has been studied well, many studies have focused on its roles in tissue/organ development. This manuscript provides new insight into the roles of Da in learning in adult brain. The manuscript would be strengthened by combining figures 5 and extended figure 5.1 into one main figure.
