# Supplementary figures and images for: The E-Protein Daughterless Regulates Olfactory Learning of Adult Drosophila melanogaster
Source: eNeuro. 2026 Jan 20;13(1):ENEURO.0051-25.2025. doi: 10.1523/ENEURO.0051-25.2025 (PMC12834652; doi:10.1523/ENEURO.0051-25.2025)

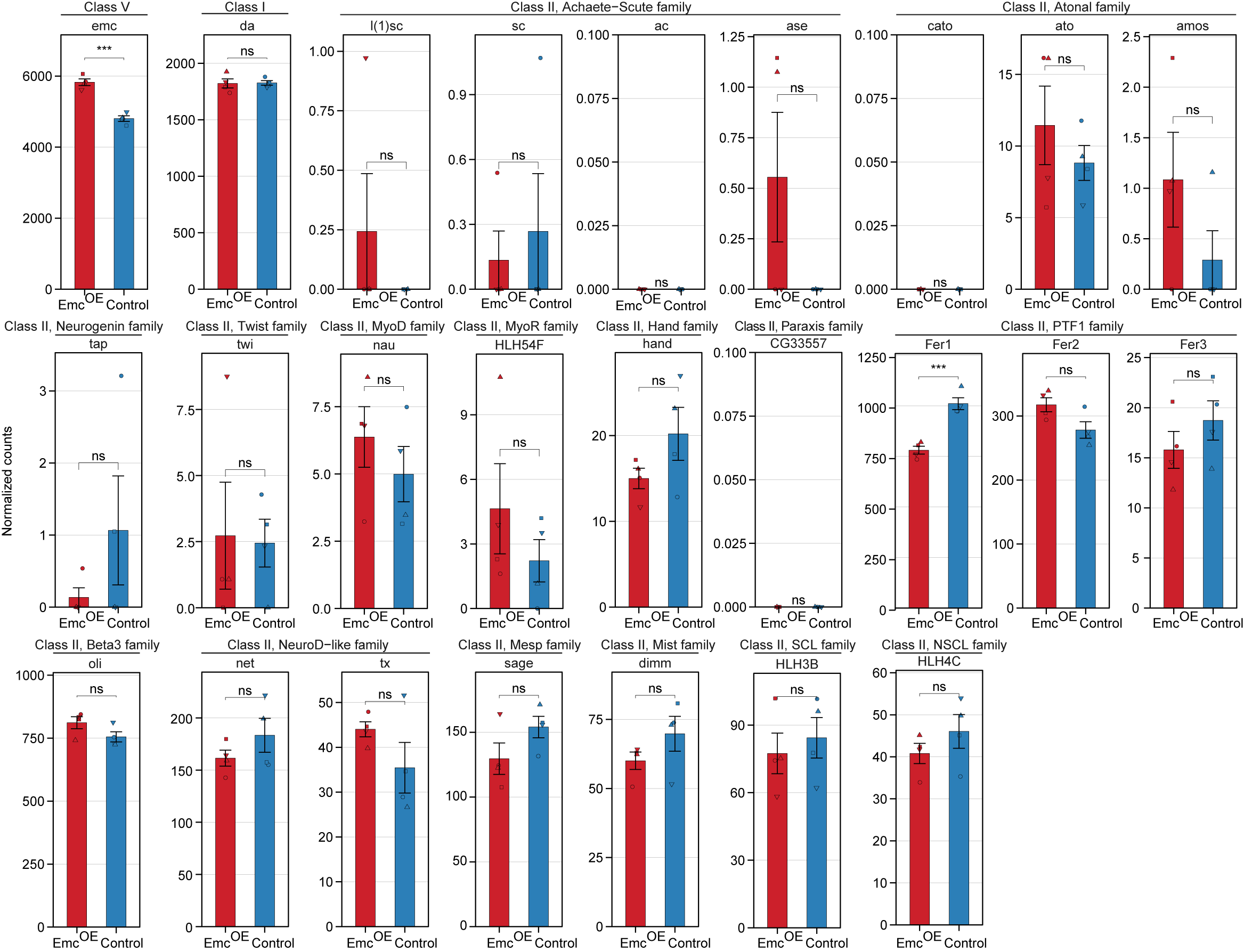

Supplement: Figure 3-2 — Expression of Da and its dimerization partners in the adult brains when Emc is overexpressed. Normalized counts of emc, da, l(1)sc, sc, ac, ase, cato, ato, amos, tap, twi, nau, HLH54F, hand, CG33557, Fer1, Fer2, Fer3, oli, net, tx, sage, dim, HLH3B and HLH4C are shown from Emc overexpression RNA-seq experiments. Classification is based on Ledent and Vervoort, 2001, and Massari and Murre, 2000. EmcOE – elavC155-Gal4 > emc, Control – elavC155-Gal4xwhite*. The replicates are shown as individual shapes and error bars represent standard error of the mean (SEM). ***p < 0.001; ns, not significant; Benjamini-Hochberg procedure (BH) -adjusted p-values. Download Figure 3-2, TIF file. [file eneuro-13-ENEURO.0051-25.2025-s003.tif]

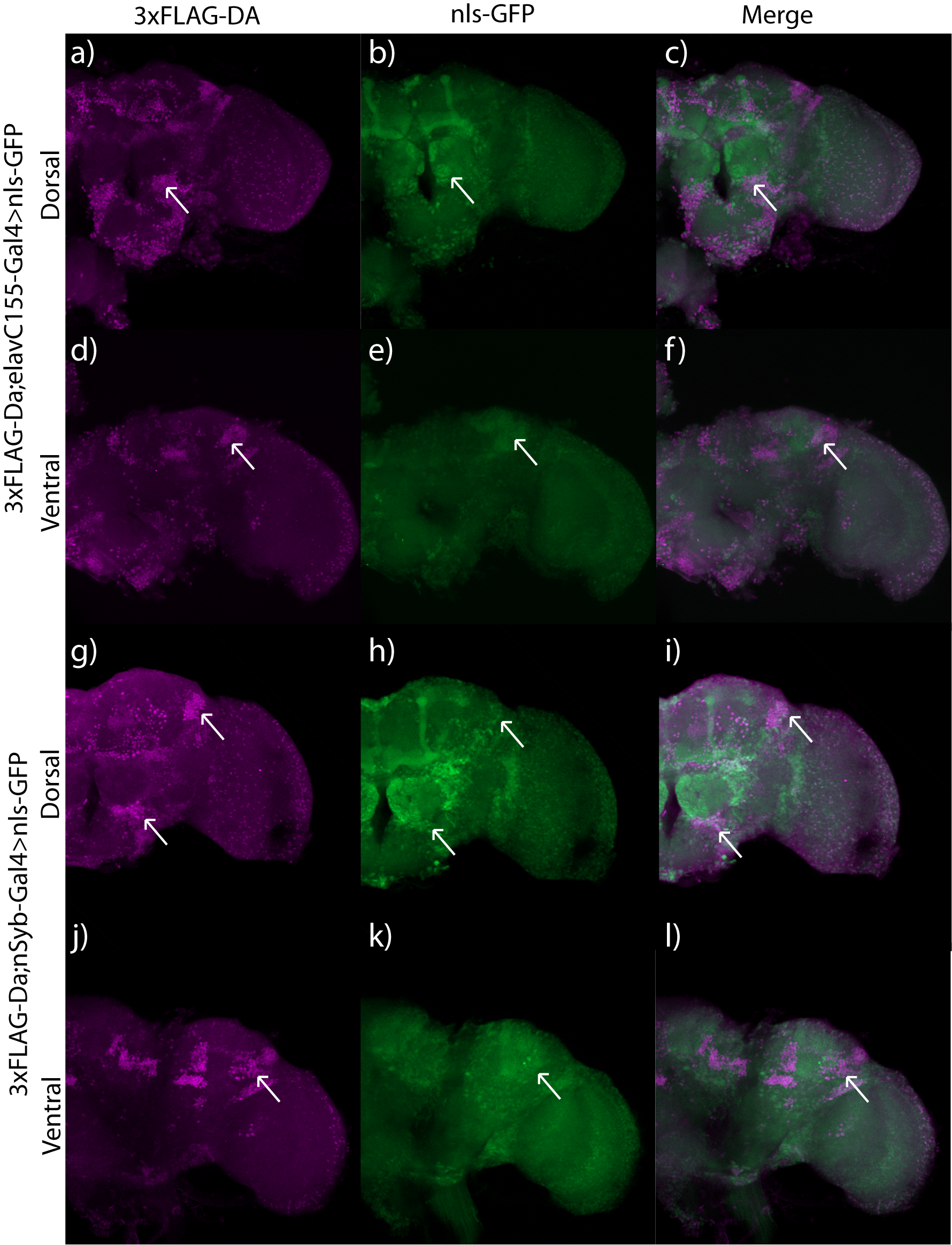

Supplement: Figure 5-1 — Expression of 3xFLAG-Da, elavC155-Gal4 and nSyb-Gal4 in the adult Drosophila brain. (a) and (g) show 3xFLAG-Da expression in the dorsal part of the brain, (d) and (j) show 3xFLAG-Da expression in the ventral part of the brain in magenta; nls-GFP expression shows the expression pattern of the drivers in green – (b) – elavC155-Gal4 dorsal part of the brain, (e) – elavC155-Gal4 ventral part of the brain, (h) – nSyb-Gal4 dorsal part of the brain, (k) - nSyb-Gal4 ventral part of the brain; on (c), (f), (i) and (l) 3xFLAG-Da and the driver’s signals are merged. White arrows point to some co-expression. Download Figure 5-1, TIF file. [file eneuro-13-ENEURO.0051-25.2025-s010.tif]
